# Preserving a legacy for our patients: The bedside-to-bench award in translational research

**DOI:** 10.1186/1479-5876-6-20

**Published:** 2008-04-22

**Authors:** Francesco M Marincola

**Affiliations:** 1 Infectious Disease and Immunogenetics Section, Department of Transfusion Medicine, Clinical Center, National Institutes of Health, Bethesda, Maryland 20854, USA

## Abstract

The *Journal of Translational Medicine *is pleased to announce a prize to recognize outstanding contributions in the field of translational medicine. This year, the Bedside-to-Bench Award was provided by an anonymous donor and supported by the *Journal of Translational Medicine *Editorial Board. Applications should be submitted directly to the *Journal of Translational Medicine *[1].

## Honoring the legacy of patients by promoting clinically-relevant, evidence-based research

"And then did we, the seven, start from our seats in horror, and stand trembling and shuddering, and aghast, for the tones in the voice of the shadow were not the tone of any one being, but of a multitude of beings, and, varying in their cadences from syllable to syllable, fell duskily upon our ears in the well-remembered and familiar accents of many thousand departed friends"

Edgar Allan Poe – "Shadow – A parable"

When a patient dies, clinical scientists have a legacy to preserve. I am reminded of parents who visited my laboratory following the death of their son; they asked the cell lines growing from his fatal cancer be named after him. Patients die in serene dignity hoping for their sole consolation that their torment was not in vain; Jonathan Gray died in the Fall of 2007 after preparing a moving editorial about his experience as a cancer patient and leaving suggestions for survivors. Recently, we published an Editorial emphasizing the need to encourage discovery-driven research as a complement to hypothesis testing to better frame experimentation in the context of disease relevance [2]. The Editorial expanded on the theme of the one that accompanied the launch of the *Journal of Translational Medicine *in which we suggested that translational research is a two way street where the bedside-to-bench direction plays a Cinderella's role [3]. The intellectual flaws of a purely hypothesis-driven approach to translational research are discussed, but less discussed are the important ethical aspects of applying discovery-driven research to clinical medicine. I believe we owe to our patients, particularly those who submit themselves to demanding clinical trials with no guaranteed benefit that their suffering shall not be wasted and their medical history shall not be forgotten. For our patients their tissues are a precious legacy to be remembered and represent their hope of saving other.

It has been almost five years since July 2003 when we launched the *Journal of Translational Medicine *as an open access journal dedicated to appropriate peer review and rapid publication of results generated by clinical investigations. The journal has since received enthusiastic international support and has been rapidly recognized and tracked/indexed by PubMed [4], PubMed Central [5], Medline [6], Thompson Scientific (ISI) [7], CAS [8], Embase [9] and Scopus [10]. The journal will receive it first official impact factor in June of 2008. This year we granted the first Excellence in Translational Medicine Award sponsored by Pfizer [11]. The award has broader goals and was granted this year by a stringent review process lead by Richard Ablin to an excellent bench-to-bedside study [12]. A second award will be dedicated to Bedside-to-Bench studies defined as those exclusively based on the study of human samples [13,14].

## The bedside-to-bench award in translational research

The recipient of The Excellence in Translational Medicine Award will receive a $5,000 prize to cover expenses for any meeting sponsored by a non-for-profit organization that is relevant to the goal of translational medicine/research. Submitted articles will be evaluated with regard to: relevance to translational medicine/research based on discovery-driven, evidence-based studies aimed at understanding human pathology by the direct study of human subjects. Selection criteria will include use of human samples not including cell lines or other tissue products expanded *in vitro *for more than one week, scientific merit, sound research design, rigorous methodology, originality and clarity.

## Eligibility criteria and selection process

• The award will be granted to the first author of a research manuscript containing original data published in the *Journal of Translational Medicine *between January 1 and December 31 of each year.

• Candidates may be self-nominated or nominated by a colleague

• Candidates must be the primary clinician or investigator involved in the project described by the article. If there are multiple first authors, each first author of the selected manuscript may claim designation as a candidate. In the event of multiple authors sharing the Award, the prize will be divided equally among them. If there are multiple first authors, this must be indicated at the time of submission.

• Authors from laboratories of the members of the selection committee are not eligible for the Award.

## Evaluation criteria

Submitted articles will be evaluated with regard to:

• Relevance to the purposes of translational medicine/research presenting discovery-driven, evidence-based studies aimed at the understanding of human pathology by the direct study of human subjects

• Scientific merit

• Sound research design

• Rigorous methodology

• Originality

• Clarity

## How to apply

All applications should be submitted to the Journal of Translational medicine – Awards Section [1].

## Selection Committee

Selection will be made by a committee of members of the Editorial Board.

We hope the Bedside-to-Bench Award together with the Excellence in Translational Medicine Award will encourage and reward those investigators who are dedicated to improving the efficiency of biomedical research with the goal of preventing and/or effectively treat human disease.
